# Synergistic Therapeutic Effects and Immunoregulatory Mechanism of Maxing Shigan Decoction Combined with Sijunzi Decoction on Viral Pneumonia in Mice

**DOI:** 10.1155/2024/2017992

**Published:** 2024-08-23

**Authors:** Huimin Huang, Huanhua Yang, Zurong Zhang, Yunlong Song, Li Li, Ke Li, Junjie Zhang, Xiaoyu Qi, Ying Wu

**Affiliations:** ^1^ Liuzhou Key Laboratory of Infection Disease and Immunology Guangxi Key Laboratory of Clinical Disease Biotechnology Research Research Center of Medical Sciences Liuzhou People's Hospital Affiliated to Guangxi Medical University, Liuzhou 545006, Guangxi, China; ^2^ School of Life Sciences Beijing University of Chinese Medicine, Beijing 102488, China

## Abstract

Influenza is defined in traditional Chinese medicine (TCM) as an epidemic febrile illness and is usually treated with herbal compound formulas under the guidance of the “Qu Xie and Fu Zheng” theories. Ma Xing Shi Gan Tang (MXSGD) is a prominent remedy for clearing heat and detoxifying toxins in the clinical treatment of influenza in TCM, playing the role of “Qu Xie.” Si Jun Zi Tang (SJZD) is recognized as one of the “Fu Zheng” formulas for strengthening the spleen and nourishing the stomach, with immunomodulatory effects. In this study, we followed the principles of “Qu Xie and Fu Zheng” to explore the effects of MXSGD combined with SJZD on viral pneumonia and its mechanism. Results showed that the couse of MXSGD and SJZD was effective in reducing the mortality rates and severity of lung pathology in lethally infected FM1 mice compared to the use of either drug alone. Moreover, further research demonstrated that the combined use suppressed TLRs and NLRP3 inflammatory signaling pathways at 4 dpi while promoting them at 7 dpi. At 10 dpi, there was a significant increase in CD11c^+^ and CD103^+^ DCs in the lungs. Together, SJZD improved the therapeutic effectiveness of MXSGD in treating influenza virus pneumonia than when used alone. MXSGD and SJZD exhibit synergistic effects in the treatment of influenza, as evidenced by the inhibition of TLR7 and NLRP3 inflammatory pathways early in the infection and facilitation of the response later. They also increase CD11c^+^ and CD103^+^ DC levels, as well as balancing Th1/Th2 cytokines.

## 1. Introduction

Severe viral infections frequently lead to serious complications or death from severe viral pneumonia. Viral pneumonia has been well described in immunocompromised patients with immune dysfunction, suggesting that the host immune plays a crucial role in the development of viral pneumonia [1]. Recent evidence showed that excessive inflammation, labeled as cytokine storm, as well as insufficient antiviral immunity, such as lymphopenia, coexisted in severe influenza A viruses (IAV) infection, and thus, a balanced immune response is required for a good clinical outcome [2, 3]. Resistance and side effects to conventional treatment drugs, including antiviral drugs such as oseltamivir, make the treatment of viral pneumonia a challenge [4]. Meanwhile, antiviral therapy for influenza viruses alone is less effective in treating the immune dysregulation caused by severe viral infection [5]. Studies are also working on immunomodulators to reduce viral-mediated inflammation flame and restore host immunity to balance [6].

Immune dysregulation is the main cause of lung injury in viral pneumonia. The coexistence of an excessive inflammatory response (inflammatory cytokine storm) and repressed antiviral immune response (e.g., delayed interferon (IFN) production or inactivated T-cell response) can exacerbate inflammation and promote virus replication leading to respiratory failure. In addition, the virus has evolved various strategies to interfere with the antiviral response in the host contributes to the poor outcome. In traditional Chinese medicine (TCM), influenza is one of the epidemic febrile diseases that were treated under the guidance of “Quxie and Fuzheng.” From the TCM point of view, “Quxie” refers to the elimination of pathogens and removal of toxins and the “Fuzheng” is interpreted as a therapy to strengthen immunity and restore damaged immunity. The roles of the two are mutually reinforcing.

Maxing Shigan decoction (MXSGD) is a classic TCM formula composed of Ephedrae Herba (*Ephedra sinica Stapf*), Armeniacae Semen Amarum (*Prunus armeniaca* L. *var. anus Maxim.*), Gypsum Fibrosum, and Glycyrrhizae Radix Et Rhizoma (*Glycyrrhiza uralensis Fisch.*). It is a representative formula in the therapeutic strategy of traditional Chinese medicine (TCM) for the treatment of COVID-19 infection and community-acquired pneumonia (CAP) [7–10]. Thus far, several drugs, including MXSGD, have demonstrated effectiveness in fighting influenza [11] and widely applied in the treatment for viral pneumonia to decrease body temperature, ameliorate headache, cough, and pharyngula [12–14]. It plays the function of heat clearing and exterior releasing, which is consistent with the therapeutic principle and method of “Quxie” in TCM. According to the protocol for diagnosis and treatment of influenza (2018), MXSGD is recommended as one of the TCM treatment strategies for influenza treatment [15]. MXSGD showed obvious anti-inflammatory effects, improved clinical symptoms, and increased the total effective rate in the patients with pneumonia. It also inhibited the invasion of pathogenic bacteria such as *Mycoplasma pneumoniae* or *Staphylococcus aureus* to protect against worsening inflammation [10, 16–18]. Animal evidence suggests that MXSGD protects LPS-induced pulmonary microvascular hyperpermeability and inflammatory response in rat models by regulating Toll-like receptors-4 (TLR4), Src, and nuclear factor kappa-B (NF-*κ*B). During viral entry, MXSGD targeted viral entry by disrupting the viral surface, inhibited IAV surface glycoprotein neuraminidase (NA) activity, and regulated phosphatidylinositol 3-kinase (PI3K)/protein kinase B (Akt) signaling against the infection [19, 20].

Sijunzi decoction (SJZD), composed of Codonopsis Radix (*Codonopsis pilosula* (*Franch*.) *Nannf.*), Atractylodis Macrocephalae Rhizoma (*Atractylodes macrocephala Koidz.*), Poria (*Poria cocos* (*Schw.*) *Wolf*), and Glycyrrhizae Radix Et Rhizoma Praeparata Cum Melle (*Glycyrrhiza uralensis Fisch.*), is a classic prescription for tonifying spleen for nourishing qi and widely applied to treat spleen deficiency [21]. Ren et al. described that the SJZD combined with Zhisou powder attenuated postinfectious cough on 45 patients and the effective rate was 91.11% [22]. Liao et al. depicted that SJZD upregulated the levels of immunoglobulins (IgA, IgG, and IgM) in serum and the frequency of CD4^+^ T cells in 43 cases of infantile with recurrent respiratory tract infection, while it downregulated the frequency of CD8^+^ T cells, following with reinforcing the immunological function [23].

Host utilizes various mechanisms to combat viruses, while both intrinsic viral pathogenicity and an excessive host innate immune response contribute to the influenza-mediated damage of the lungs [24]. Thus, the balance between tissue tolerance and immune resistance is critical to viral challenge. Toll-like receptor-7 (TLR7) signaling has stimulated the expression of proinflammatory cytokines and type I interferons (IFNs) against the virus by activating NF-*κ*B and interferon-regulatory factor 7 (IRF7) via the adaptor myeloid differentiation primary response gene 88 (MyD88) [25]. NOD-like receptor thermal protein domain-associated protein 3 (NLRP3) inflammasome induced the proteolytic processes of pro-interleukin (IL)-18 and pro-IL-1*β* and protected mice against influenza virus via triggering pyroptosis and IL-1*β*-mediated neutrophil recruitment [26–28]. In adaptive immunity, CD4^+^ T cells and CD8^+^ T cells play an important role in the immune response and virus clearance. After infection with influenza virus (A/Puerto Rico/8/1934 H1N1, PR8), the levels of CD4^+^ T and CD8^+^ T cells were decreased [29]. The migration of CD11c^+^ DC cells from lungs to mediastinal lymph nodes was reduced, and the level of CD103^+^ DC cells was lower than that in the control group. The balance of cytokines derived from T helper 1 (Th1) cells and T helper 2 (Th2) cells also plays an important role in maintaining moderate immunoreactions [30]. Liu et al. described that the contents of tumor necrosis factor *α* (TNF-*α*) and interferon-*γ* (IFN-*γ*) were obviously upregulated, while the level of IL-4 was not significantly changed in influenza virus H1N1 (A/FM/1, FM1)-infected mice and the pathological process of FM1 infection might be correlated with excessive inflammation mediated by proinflammatory cytokines [31].

The flu or flu-like illness is often accompanied by cough, runny nose, sore throat, headache, fever, diarrhea, muscle or joint pain, or fatigue. When infected, mice show loss of appetite, inactivity, ruffled fur, a hunched posture, hypothermia, disturbance of the intestinal flora, and respiratory distress, which is consistent with the syndrome of qi deficiency in TCM and should adopt the treatment strategy of “Fuzheng.” Sijunzi decoction (SJZD) is one of the most accepted “Fuzheng” formulae for strengthening and nourishing the spleen. Therefore, we attempted to combine MXSGD and SJZD based on TCM theories to investigate whether their conjunction could enhance the efficacy. Therefore, we aimed to investigate how MXSGD combined with SJZD would affect efficacy in FM1-infected mice. The physical sign changes and lung tissue damage conditions in the mice were evaluated. The pulmonary mRNA and protein levels of TLRs and NLRP3 inflammasome signal pathways were determined. The number of T cells and DCs cells and the cytokine levels of IL-18, IL-1*β*, TNF-*α*, IFN-*γ*, IL-4, and IL-10 were measured. These results would provide evidence for applying the theory of “Quxie” and “Fuzheng” in the treatment of viral pneumonia, and clarify the mechanism of the anti-inflammation and immunoregulatory effect of MXSGD combined with SJZD. To discuss the application of TCM theory in drug use and treatment is of great significance to clinical diagnosis and rational treatment.

## 2. Materials and Methods

### 2.1. Preparation of MXSGD and SJZD

All herbs were provided by Beijing Tongrentang Pharmaceutical Co., Ltd., China. For the preparation of MXSGD, Armeniacae Semen Amarum (22.5 g), Gypsum Fibrosum (60 g), and Glycyrrhizae Radix Et Rhizoma (15 g) were soaked in distilled water for 1 h and boiled for 15 min (100°C); then, Ephedrae Herba (15 g) was added and boiled for 5 min; and then, the residue was filtered and re-extracted. The filtrate was concentrated to 1.5 g/ml. For the preparation of SJZD, Codonopsis Radix (25 g), Atractylodis Macrocephalae Rhizoma (25 g), Glycyrrhizae Radix Et Rhizoma Praeparata Cum Melle (25 g), and Poria (25 g) were extracted twice at 100°C for 20 min and then concentrated to 1.5 g/ml. Their combination was prepared by mixing 30 ml MXSGD and SJZD 30 ml. The mixture was then concentrated to 30 ml. Decoction samples were dried by rotary evaporator and stored in a cool and dark place. The detail information of the herbs is shown in Table 1.

### 2.2. UPLC-Q-Orbitrap HRMS/MS Analysis

The LC-MS analysis was performed using UPLC-Q-Orbitrap HRMS-MS system (Thermo Fisher, USA) equipped with an ACQUITY UPLC HSS T3 column (2.1 × 100 mm, 1.8 *μ*m) at 35°C. Mobile phases consisted of water (A) and acetonitrile (B) (both containing 0.1% formic acid), with a gradient elution of 0–10 min, 0–30% B; 10–25 min, 30–40% B; 25–30 min, 40–50% B; 30–40 min, 50–70% B; and 30–40 min, 50–70% B, at a flow rate of 0.2 ml/min. The injection volume was 5 *µ*L. The MS acquisition was performed on Q-Exactive Orbitrap HRMS/MS in positive and negative ionization mode. Sheath gas flow was 40/45 arb; aux gas flow was 11/10 arb; spray voltage was 3.5 kV for positive ionization and 3.2 kV for negative ionization; the capillary temperature was 350°C; and Aux gas heater temperature was 220°C.

### 2.3. Animals and Viruses

Six- to eight-week-old specific pathogen free (SPF) male BALB/c mice (16–20 g) were purchased from Beijing Vital River Laboratory Animal Technology Company (SCXK20070001, Beijing, China) and housed in an SPF laboratory animal room with 12-hr light/dark cycle. All animal treatment and experiments were overseen and approved by the experimental animal ethics committee of the Beijing University of Chinese Medicine (Permit number: BUCM-4-2021030701-1102) and Liuzhou people's hospital (Permit number: LRYIACUC2024015). To euthanize animals, CO_2_ inhalation was used at the end of the experiment before cervical dislocation.

To evaluate the antivirus therapeutic efficacy, 85 mice were randomly divided into six groups (*n* = 15 per group, except normal control group): normal control group (NC, *n* = 10), FM1 group, MXSGD group, SJZD group, MXSGD + SJZD group, and oseltamivir group. Mice were challenged with a lethal dose of FM1, and the survival rate, animal bodyweight change, temperature, etc., were recorded and lung tissues were collected.

For mechanism research, 90 mice were randomly divided into six groups (*n* = 15 per group): the abovementioned group setting. Mice were challenged with virus (a dose of LD_50_), and qPCR analysis of mRNA extracted from mouse lung tissue (*n* = 5 mice/group) was conducted at 4 and 7 dpi. The flow cytometric analysis was performed in 7 and 10 dpi.

The FM1 virus strain was provided by the Institute of Virology, Chinese Academy of Preventive Medicine (Beijing, China), and kindly donated by Prof. Cui Xiaolan from the Institute of Chinese Materia Medica China Academy of Chinese Medical Sciences (Beijing, China). The virus was propagated in the allantoic cavities of 9-day-old SPF embryonic chicken eggs at 37°C for 48 h followed by preservation at −20°C for 1 h and transfer at 4°C overnight. The virus-containing allantoic fluid was harvested [32]. The LD_50_ of FM1 in mice determined by the Reed–Muench method was calculated to be 10^−4.4^/0.02 ml. Virus stocks were collected and stored at −80°C.

### 2.4. Survival Rate Analysis

Mice were anesthetized and inoculated intranasally with 25 ul of virus solution (lethal dose of FM1 strain). The survival rates of all mice were evaluated, and the weight changes, body temperature, clinical signs of mice, and the number of deaths were recorded.

### 2.5. Lung Histology

Lungs from mice uninfected or infected with FM1 were dissected, fixed in 10% phosphate-buffered formalin, embedded in paraffin, sectioned, stained with hematoxylin and eosin, and then observed by light microscopy to observe morphologic changes.

### 2.6. Establishment of FM1 Pneumonia Mouse Model

The mice were infected intranasally with LD_50_ of FM1. Infection mice were randomly divided into four groups: MXSGD, SJZD, MXSGD + SJZD, and oseltamivir (2.5 mg/ml, 0.9% NaCl), which was given twice a day through the intragastrical at 1 h after the virus infection following the treatment schedule for each group.

### 2.7. Western Blot (WB) Assay

The expression of p-NF-*κ*B (#3031, Cell Signaling Technology) and cysteinyl aspartate specific proteinase-1 (caspase-1) (SAB4503271, SIGMA) was detected by WB. Proteins from lung tissues were extracted with lysis buffer and quantitated by bicinchoninic acid (BCA) assay according to the manufacturer's protocol. Lysate samples were separated by SDS-polyacrylamide gels (SDS-PAGE) and transferred to polyvinylidene difluoride (PVDF) membranes, blocked with 5% defatted milk on shaker for 1 h at room temperature, and incubated with diluted antibodies at 4°C overnight. The corresponding secondary antibody (1 : 2000 diluted) was incubated at room temperature for 1 h, prior to detection with enhanced chemiluminescence (ECL) technique, and analyzed by Quantity One imaging system.

### 2.8. ELISA

Serum levels of IL-18 in mice were measured according to the manufacturer's instructions (Abnova, Taipei, China).

### 2.9. Quantitative Real-Time PCR (RT-qPCR)

Trizol reagents (Thermo Fisher Scientific, USA) were used to isolate total RNA from lung tissues. RNA was reverse-transcribed into first-strand cDNA using Verso cDNA Synthesis Kit (ThermoFisher, Waltham, USA). SYBR® Green Realtime PCR Master Mix (Toyobo, Osaka, Japan) was carried out for RT-qPCR analysis. The primers were synthesized by Sangon Biotech (Shanghai, China), and the details are shown in Table 2. The relative quantification of expressive genes was processed using the 2^−ΔΔCt^ method.

### 2.10. Flow Cytometry

100 mg lung tissues from each mouse were minced, digested, and centrifuged for single-cell isolation. 45 *µ*L microspheres were plated into a 96-well plate. Diluent and 45 *µ*L of cells were then added and allowed to shake for 1 h at room temperature in the dark. 25 *µ*L of 1 × Biotin-dAb (BD Pharmingen, USA) of IL-1*β*, TNF-*α*, IFN-*γ*, IL-4, or IL-10 was then added and allowed to shake for 30 min in the dark and quantified by flow cytometer.

CD4^+^, CD8^+^ T cells and CD11c^+^, CD103^+^ DC cells in lung tissues collected in 7 and 10 dpi were detected in the same procedure. The antibody of CD4, CD8, CD11c, and CD103 was purchased from BD Pharmingen (BD, USA).

### 2.11. Statistical Analysis

All data analyses and the results are expressed as the mean ± standard deviation (SD) by using GraphPad software (version 9.0). Multiple group comparisons were performed using one-way analysis of variance (ANOVA), followed by Dunnett's test to determine significant differences from the control. Differences were considered significant with a *p* value <0.05.

## 3. Results

### 3.1. Identification of the Chemical Composition of MXSGD, SJZD, and Their Combination

**Figure 1:**
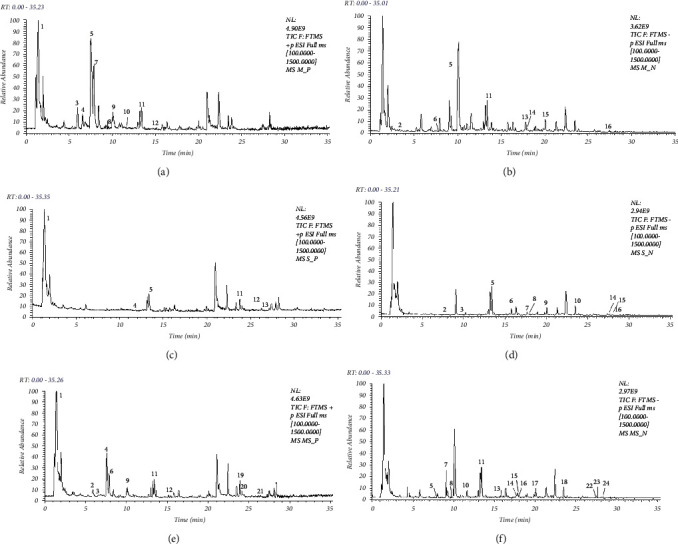
Total ion chromatograms (TIC) of MXSGD, SJZD, and MXSGD + SJZD in positive and negative ion modes were characterized using UPLC-Q-Orbitrap HRMS-MS. Positive ion mode and negative ion mode of (a, b) MXSGD, (c, d) SJZD, and (e, f) MXSGD + SJZD.

The base peak chromatograms of MXSGD, SJZD, and their combination in positive and negative ion mode are shown in Figures 1(a), 1(b), 1(c), 1(d), 1(e), and 1(f), respectively. Preliminary characterization of 16, 16, and 24 compounds was carried out in combination with the plots and based on standard database matches and literature information (Tables S1–S3).

### 3.2. MXSGD + SJZD Improved Symptoms and Alleviated Clinical Signs, Attenuated Pathological Changes, and Ameliorated FM1 Infection

**Figure 2:**
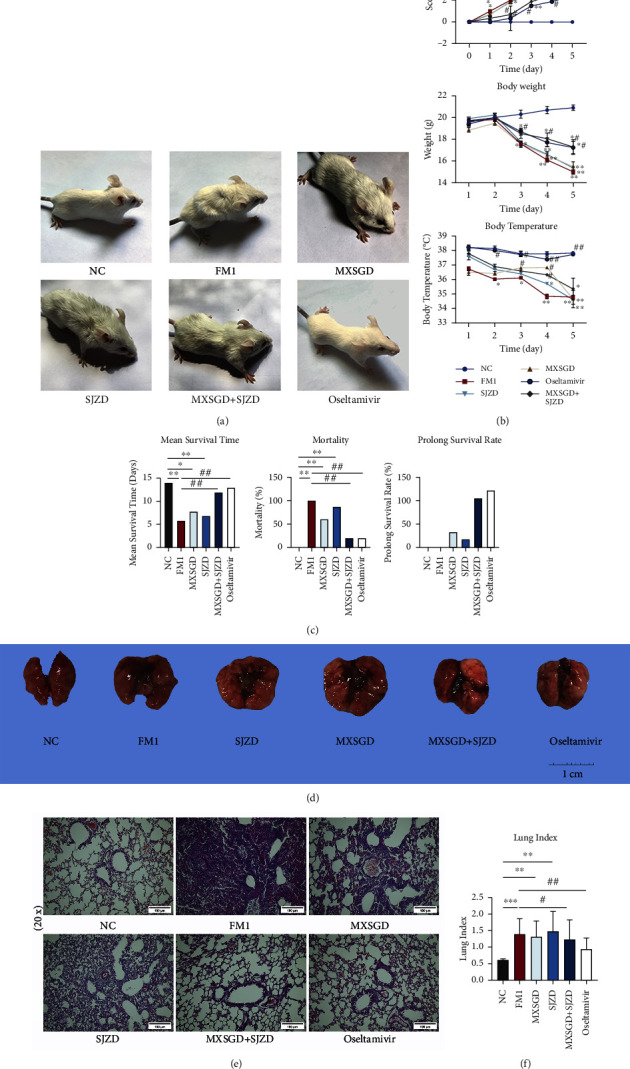
The effect of MXSGD + SJZD on symptoms, clinical signs, and pulmonary functions. (a) Mice physical characteristics. (b) The score of clinical signs was evaluated. 0: healthy; 1: barely ruffled fur; 2: ruffled fur, but active; 3: ruffled fur and inactive; 4: ruffled fur, inactive, hunched, and gaunt; 5: dead. Body weight and body temperature changes of mice were measured and evaluated. (c) The mean survival time, mortality, and prolong survival rate of mice. (d) Morphological changes of lung tissues. (e) Hematoxylin-eosin staining of lung tissues (20×), scale bar = 100 *μ*m. (f) The lung index of mice. The FM1 group and treatment groups, *n* = 15; normal control (NC) groups, *n* = 10. ^∗^*P* < 0.05 or ^∗∗^*P* < 0.01 or ^∗∗∗^*P* < 0.001 vs. NC group; ^#^*P* < 0.05 or ^##^*P* < 0.01 vs. FM1 group.

We compared the clinical signs and pneumonia lesions of each group. The normal control group mice had no change in the general appearance. FM1-infected mice showed significant flu-like symptoms, such as fur puckering, immobility, slow movement, hunching, emaciation, hunched back, and even death. The symptoms of the MXSGD or SJZD group were slightly better than those of the FM1 group, while MXSGD + SJZD and oseltamivir treatment could restore milder clinical symptoms after infection (Figure 2(a)). The infection mice also lost significant weight and became hypothermic. The MXSGD + SJZD combination was effective in alleviating the flu-like symptoms and the mice showed significant recovery in body weight and body temperature compared to mice in the FM1 group (Figure 2(b)). Compared to FM1-infected mice, treatment with the MXSGD + SJZD combination had lower mortality rates, longer mean survival times, and higher prolonged survival rates (Figure 2(c)).

Influenza A virus (IAV) is a major causative agent of lower respiratory tract infections, and infection with FM1 can lead to a severe inflammatory response in the lungs. Lung tissue from mice in the NC group appeared bright pink with a smooth surface and a soft, elastic texture. FM1-infected mice showed marked lung lobe swelling and hemorrhage (Figure 2(d)). Pathological sections also showed irregular and disrupted alveoli, thickened alveolar septa, infiltration of inflammatory cell aggregates and abundant secretions, and congestion in the bronchial lumen of FM1-infected mice (Figure 2(e)). That was accompanied by a marked upregulation of the lung index (Figure 2(f)). In contrast, the combination of MXSGD + SJZD effectively improved congestion and hemorrhage in lung tissue, reduced inflammatory cell infiltration, inhibited plasma exudation in the bronchial lumen, and reduced damage to alveolar or capillary endothelial cells. To some extent, it also ameliorated pathological changes in the lungs and downregulated the lung index (Figures 2(d) and 2(f)).

### 3.3. TLR and NLRP3 Inflammasome Signal Pathways in FM1-Infected Mice Were Inhibited by MXSGD + SJZD at 4 dpi and Promoted at 7 dpi

**Figure 3:**
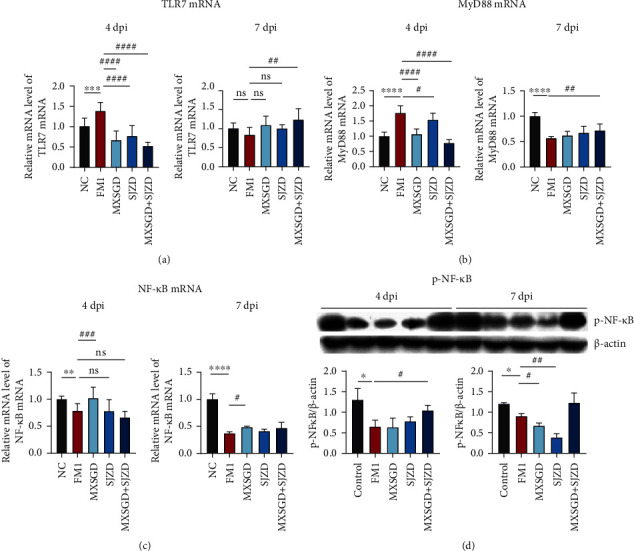
Effect of MXSGD + SJZD on TLR signaling at 4 and 7 dpi (*n* = 5). (a) TLR7, (b) MyD88, (c) NF-*κ*B mRNA levels, and (d) p-NF-*κ*B protein change in lung tissue. ^∗^*P* < 0.05 or ^∗∗^*P* < 0.01 or ^∗∗∗^*P* < 0.001 vs. NC group; ^#^*P* < 0.05 or ^##^*P* < 0.01 vs. FM1 group.

After establishing the mouse FM1 infection model with LD_50_, the expression of the TLR7 pathway was altered. The pulmonary mRNA expression of TLR7, MyD88, and NF-*κ*B was abnormally upregulated in FM1-infected mice at 4 dpi and was decreased at 7 dpi (Figure 3). MXSGD + SJZD inhibited these mRNAs and protein expression on day 4 and promoted them on day 7. The TLR7 pathway might be activated that p-NF-*κ*B showed an increased trend after treatment with MXSGD + SJZD.

**Figure 4:**
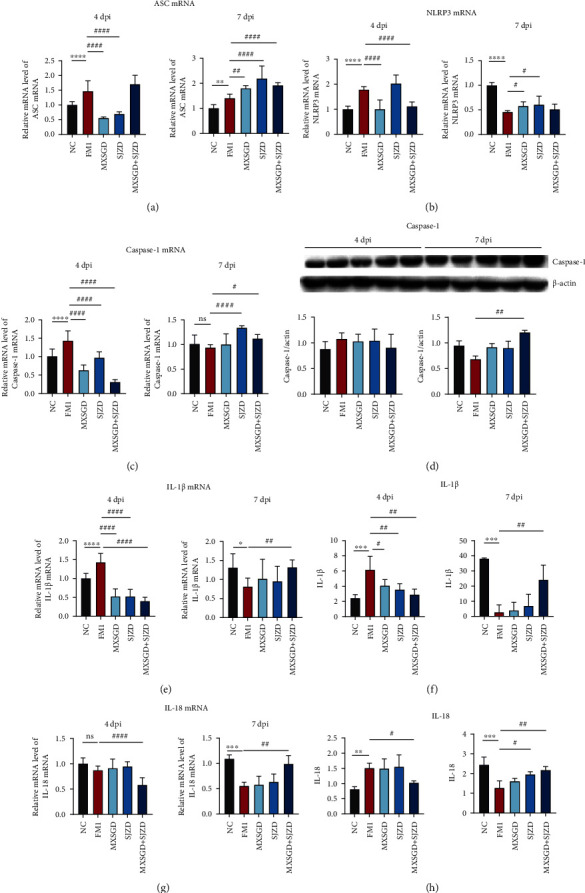
Effect of MXSGD + SJZD on the NLRP3 inflammasome pathway at 4 and 7 dpi (*n* = 5). (a) ASC, (b) NLRP3, (c) caspase-1 mRNA, and (d) caspase-1 protein in lung tissues. (e) IL-1*β* mRNA, (f) IL-1*β*, (g) IL-18 mRNA, and the level of (h) IL-18 in lung tissues was measured. ^∗^*P* < 0.05 or ^∗∗^*P* < 0.01 or ^∗∗∗^*P* < 0.001 vs. NC group; ^#^*P* < 0.05 or ^##^*P* < 0.01 vs. FM1 group.

The disruption of NLRP3 pathways was observed by FM1 infection at 4 dpi and inhibited at 7 dpi. The disruption of pathway proteins was significantly regulated by MXSGD + SJZD (Figure 4). The levels of IL-1*β* and IL-18 in the supernatant confirmed the same change, and MXSGD + SJZD had the most significant efficacy (Figures 4(f) and 4(h)).

### 3.4. Levels of CD11c^+^ and CD103^+^ DC in Lung Tissue Were Significantly Upregulated by MXSGD Combined with SJZD at 10 dpi

**Figure 5:**
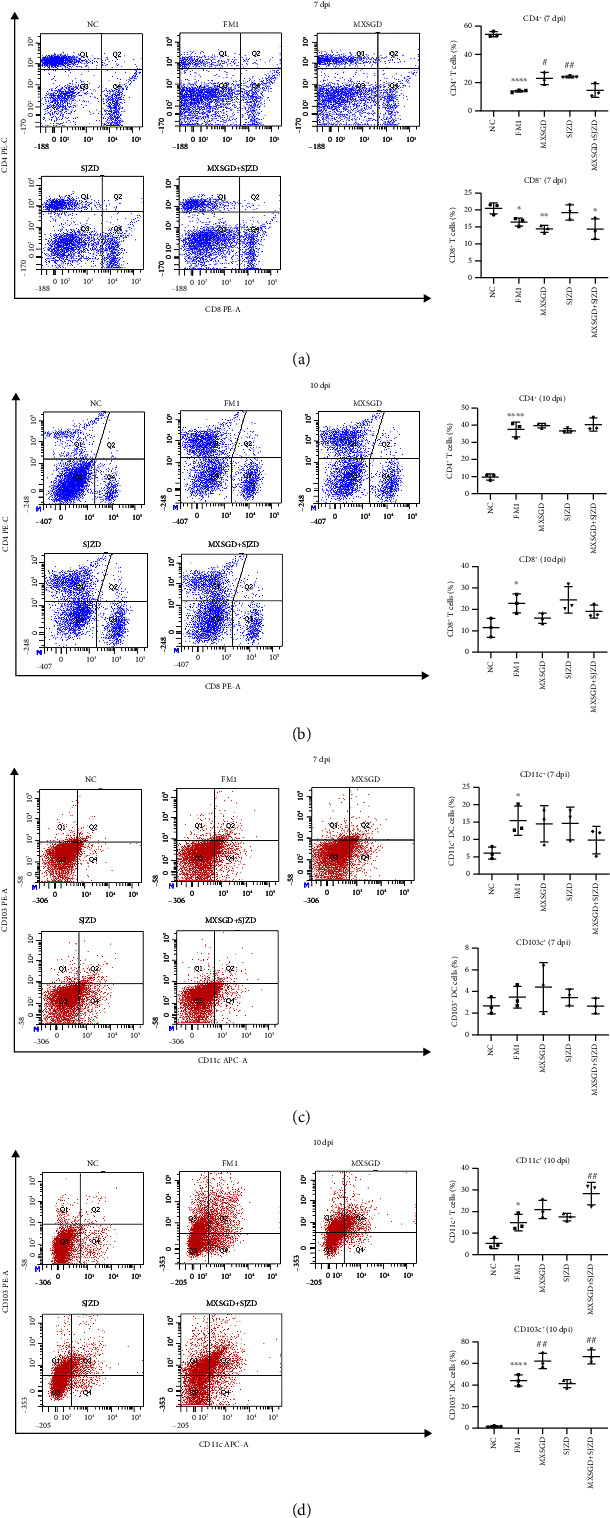
Flow cytometry results of T cell and DC cell in lung tissues of each group on day 7 and day 10 (*n* = 3). CD4^+^ and CD8^+^ T-cell levels change on (a) day 7 and (b) day 10. CD11c^+^ and CD103^+^ DC cell levels change on (c) day 7 and (d) day 10. ^∗^*P* < 0.05 or ^∗∗^*P* < 0.01 or ^∗∗∗^*P* < 0.001 vs. NC group; ^#^*P* < 0.05 or ^##^*P* < 0.01 vs. FM1 group.

To investigate the immune cell response in the lungs of infected mice, we detected changes in the number of T cells and DCs cells in lung tissue using flow cytometry. Compared to the normal group, the number of CD4^+^ and CD8^+^ cells in the lung tissue of infected mice reduced at 7 dpi and increased at 10 dpi (Figures 5(a) and 5(b)). Virus infection increased the number of T cells, but MSXGD or SJZD treatment has no significant effect. On the contrary, the levels of CD11c^+^ and CD103^+^ DC increased in infected mice from day 7 to day 10 (Figures 5(c) and 5(d)). After the treatment with MXSGD + SJZD, CD11c^+^ levels were significantly increased at 10 dpi. The number of CD103^+^ DC cells in the MXSGD and combination groups was significantly higher than that in the virus group. Therefore, MXSGD + SJZD cannot directly increase the number of T cells, and its mechanism may involve increasing the antigen presentation and enhancing the activity of T cells.

### 3.5. The Balance of Th1/Th2 Cell Cytokines Was Modulated by MXSGD Combined with SJZD

**Figure 6:**
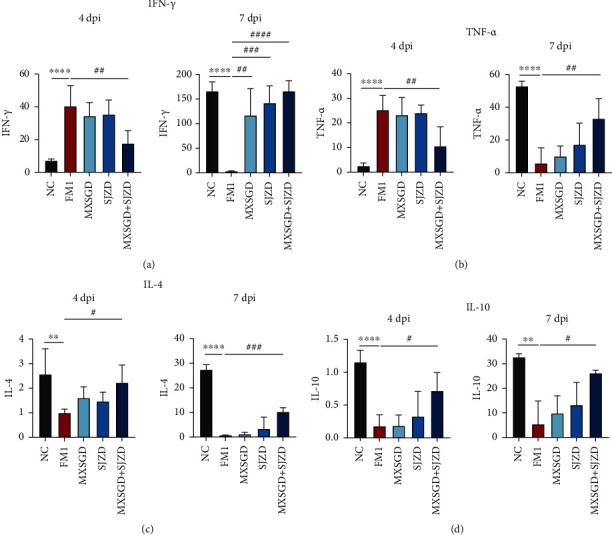
The effect of MXSGD + SJZD on Th1/Th2 cytokines (*n* = 5). (a) IFN-*γ*, (b) TNF-*α*, (c) IL-4, and (d) IL-10 in lung tissues were measured. ^∗^*P* < 0.05 or ^∗∗^*P* < 0.01 or ^∗∗∗^*P* < 0.001 vs. NC group; ^#^*P* < 0.05 or ^##^*P* < 0.01 vs. FM1 group.

The proinflammatory factors of IFN-*γ* and TNF-*α* were upregulated in FM1-infected mice at 4 dpi by flow cytometry detection, while suppressed at 7 dpi (Figures 6(a) and 6(b)), while the anti-inflammatory cytokines (IL-4 and IL-10) change in the opposite direction (Figures 6(c) and 6(d)). MXSGD + SJZD has the best effect of inhibiting inflammation in the early stage and promoting an inadequate response in the late stage by regulating the Th1/Th2 ratio.

## 4. Discussion

Flu is one of the epidemic febrile diseases in traditional Chinese medicine, which was treated under the guidance of “Quxie” and “Fuzheng.” MXSGD, first created by Zhang, Zhongjing, is a classic traditional Chinese formula for relieving cough and asthma, eliminating phlegm and clearing heat. SJZD, a formula associated with “Fuzheng” with the efficacy of “invigorating spleen and replenishing qi,” could be used in spleen-qi deficiency syndrome to improve immunity. FM1-infected mice showed wrinkled fur, inactivity, slowness, and hunchback, which were consistent with the description of lung qi deficiency syndrome in TCM. According to the theory of TCM, in the treatment of external contraction diseases, while “Quxie” to eliminate pathogens, we should also pay attention to “Fuzheng” to strengthen healthy qi. Therefore, MXSGD + SJZD was used to treat infected mice in this study.

In our study, SJZD did not have a significant effect on pneumonia, but it was a significant enhancer of the therapeutic effect of MXSGD. When infected with a lethal dose of FM1 virus, MXSGD reduced mortality and prolonged mean survival in mice when combined with SJZD, and improved prolonged survival more effectively than the drug alone. Moreover, the combination of MXSGD and SJZD reduced lung indices and markedly improved the extent of lung tissue damage. Taken together, these results suggest a significant synergistic effect of MXSGD in combination with SJZD. We then investigated the mechanism underlying the effect of MXSGD combined with SJZD in viral pneumonia. Evidence showed that excessive inflammation, referred to as cytokine storm, and insufficient antiviral immunity, such as lymphopenia, coexist in severe IAV infection, and thus, a balanced immune response is required for a good clinical outcome. Excessive or prolonged activation of the TLRs pathway is strongly associated with acute lung injury and harmful inflammation in the early stages of viral infection [33, 34]. This leads to the release of inflammatory factors. Thus, regulation of the inflammatory responses of the TLRs signaling pathway may alleviate acute lung injury induced by influenza virus and may be an effective therapy for influenza infection. In this study, we provide evidence that MXSGD + SJZD significantly downregulated the TLR7 pathway at 4 dpi and activated it at 7 dpi in response to FM1 infection. At the early stage of immune infection, MXSGD + SJZD can control the inflammatory damage caused by infection by inhibiting the TLR7 pathway. On day 7, MXSGD combined with SJZD can activate the TLR pathway during viral replication and promote an antiviral adaptive immune response in vivo.

**Figure 7:**
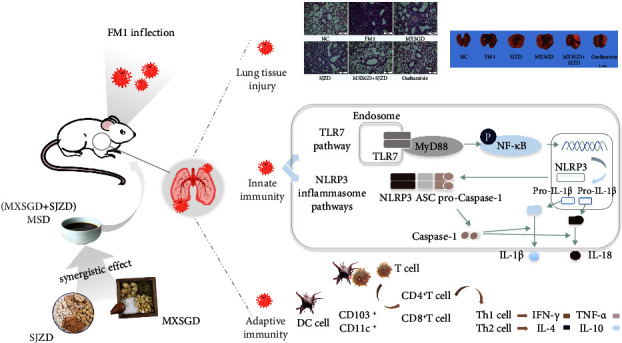
Mechanism of MXSGD and SJZD conjugated for the treatment of influenza-infected mice.

The NLRP3 inflammasome is an essential component of the host immune response to influenza virus infection. However, excessive activation of the NLRP3 inflammasome pathway may have pathological implications in influenza virus infection [35]. Therefore, the immunological responses of the NLRP3 inflammasome pathway must be strictly modulated to avoid hyperinflammatory or immunocompromised states during influenza infection. Our results showed that MXSGD + SJZD could inhibit the hyperinflammatory state mediated by the NLRP3 inflammasome pathway at 4 dpi and maintain the activity of the pathway and the release of certain inflammatory factors conducive to virus clearance at 7 dpi. In contrast, influenza mice treated with MXSGD and SJZD alone were unable to regulate the dysregulation of this pathway. CD4^+^ and CD8^+^ T cells have an important role to play in the protective immune response to an influenza infection. Under attack by influenza virus, CD4^+^ T cells are activated after recognizing viral epitopes associated with MHC class II molecules and interacting with costimulatory molecules on APC. Initial CD8^+^ T cells are activated after recognizing viral epitopes associated with MHC class I molecules on APC in draining lymph nodes and then differentiate into CTL cells that migrate to the site of infection and play a role in recognizing and removing infected cells. Timely immune response plays a crucial role in the process of viral infection. The results showed that MSXGD and SJZD did not affect the number of CD4^+^ and CD8^+^ in FM1-infected mice (Figure 5). DCs are the most efficient antigen-presenting cells. After viral infection, CD11c^+^ DC was transported and aggregated in draining lymph nodes, where they progressively matured and promoted the differentiation of primary lymphocytes. CD103^+^ DC, a migratory DC subset, can secrete complements C3 and C5, resulting in the induction of T-cell immune responses and virus clearance after influenza infection [36, 37]. It is highly sensitive to influenza infection and highly effective at activating lymph node cells, which is critical for initiating CD8^+^ T-cell responses early in infection [38–41]. Interestingly, although the drug had no effect on T-cell numbers, MXSGD and MXSGD + SJZD could gradually promote DC cell antigen presentation in the lung tissue of influenza-infected mice. The combination of SJZD can enhance the effect of MXSGD in enhancing antigen presentation and promoting T-cell immunity. In healthy people, the frequencies of Th1/Th2 cells are relatively balanced, which is important for maintaining normal immune function, and the delicate balance of Th1/Th2 responses is disrupted in IAV infection [42, 43]. Th1 cell polarization predominates, IFN-*γ* secretion predominates, and macrophage infiltration is excessive in FM1-treated mice [31]. IL-4 production is significantly reduced in the serum and bronchoalveolar lavage fluid of infected mice, while IFN-gamma release is increased [44]. On the other hand, Th1 cells modulate cellular immunity, while Th2 cells regulate humoral immunity [45]. Isotype switching to IgE has been induced by IL-4 in B cells [46]. However, Th2 cell-mediated allergic airway disease can be induced by a Th1-mediated antiviral response to influenza virus infection [47]. Therefore, the balance of Th1/Th2 cell cytokines should be carefully regulated during influenza virus infection. In the present study, MXSGD + SJZD may enhance the antiviral effect by modulating the immune response by promoting the expression of Th1 cell-derived TNF-*α* and IFN-*γ* and Th2 cell-derived IL-4 at a late stage of FM1 infection. The upregulated expression of Th2 cell-derived IL-10 may suppress the immunological dysfunction of Th1 cells in MXSGD combined with SJZD-treated mice at a late stage of FM1 infection (Figure 7).

Influenza is one of the epidemic febrile diseases. The treatment principle of “Quxie” and “Fuzheng” is applied throughout the process of influenza prevention and treatment to improve the prognosis of patients. The infected mice show the symptoms of “qi deficiency,” such as loss of appetite, inactivity, and ruffled fur, which are treated by SJZD based on the “Fuzheng” strategy. However, the symptoms of viral infection in the host will be more complex. Therefore, the method of “Fuzheng” should be adopted according to different clinical manifestations.

In modern medicine, oseltamivir, one of the classic antiviral drugs, is widely used to treat influenza virus pneumonia and was selected as the positive drug in this study. Early treatment of viral pneumonia is critical, as our data show that in a mouse model of lethal dose infection, mice receiving antiviral therapy at the time of virus entry (1 dpi) still have high survival rates. However, the reality is more complicated because antiviral treatment is often not available in the early stages of the virus. It is therefore necessary to consider combination treatment with antivirals and other drugs. Our results will provide clues and ideas for drug therapy of viral pneumonia in the clinic.

## 5. Conclusion

In conclusion, our study showed that the combination of MXSGD and SJZD was more effective than either MXSGD or SJZD alone in FM1 influenza inflection. The synergistic effects include improvement in clinical signs, weight gain, and body temperature in mice. In addition, MXSGD combined with SJZD plays a beneficial role in activating a balanced inflammatory response in the host to limit immunopathological damage and improve clinical and survival outcomes. They may facilitate antiviral immunological responses in the late stage of FM1 infection by regulating TLRs and NLRP3 inflammasome pathways, potentiating the regulation of CD11c^+^ and CD103c^+^ and maintaining the balance of Th1/Th2 cytokines. The theory of “Quxie and Fuzheng” is reflected in the combination of MXSGD and SJZD and confirmed to improve the curative effect of antiviral pneumonia, which may provide an effective strategy for future clinical treatment.

## Figures and Tables

**Table 1:** Herbs information of Maxing Shigan decoction and Sijunzi decoction.

Formula	Latin name	English name	Chinese name
Maxing Shigan decoction	*Prunus armeniaca* L*. var. ansu* Maxim.	Armeniacae Semen Amarum	Kuxingren
	*Gypsum Fibrosum*	Gypsum Fibrosum	Shigao
	*Glycyrrhiza uralensis* Fisch.	Glycyrrhizae Radix et Rhizoma	Gancao
	Ephedra sinica Stapf	Ephedra herb	Ma Huang

Sijunzi decoction	*Codonopsis pilosula (Franch.*) Nannf.	Codonopsis Radix	Dang Shen
	*Atractylodes macrocephala* Koidz.	Atractylodis Macrocephalae Rhizoma	Bai Zhu
	*Glycyrrhiza uralensis* Fisch.	Glycyrrhizae Radix et Rhizoma Praeparata Cum Melle	Zhigancao
	*Poria cocos (Schw.*) Wolf	Poria	Fuling

**Table 2:** Primer sequences for RT-qPCR assay.

No.	Genes	Direction	Sequence
1	*TLR7*	Forward	5′-CCCAGAAAATGTCCTCAACAAT-3′
		Reverse	5′-AACCCACCAGACAAACCACAC-3′

2	*MyD88*	Forward	5′-TACAGGTGGCCAGAGTGGAA-3′
		Reverse	5′-GCAGTAGCAGATAAAGGCATCGA-3′

3	*NF-κB*	Forward	5′-ACCACTGCTCAGGTCCACTGTC-3′
		Reverse	5′-GCTGTCACTATCCCGGAGTTCA-3′

4	*ASC*	Forward	5′-GGCCAGAAGCACAAACTCATC-3′
		Reverse	5′-ACACCAAAGAGCCACAGAACA-3′

5	*NLRP3*	Forward	5′-TCTCTCCCGCATCTCCATTT-3′
		Reverse	5′-GCTGTCCCGCATTTTAGTCC-3′

6	*Caspase-1*	Forward	5′-ACTCGTACACGTCTTGCCCTC-3′
		Reverse	5′-CTGGGCAGGCAGCAAATTC-3′

## Data Availability

All raw data and graphs are available upon request.

## Supplementary Materials

Supplementary Materials Characterization of compounds identified from MXSGD, SJZD, and MXSGD + SJZD by UPLC-Q-Orbitrap-MS, shown in Tables S1, S2, and S3.
