# Supplementary material for: Synergistic Therapeutic Effects and Immunoregulatory Mechanism of Maxing Shigan Decoction Combined with Sijunzi Decoction on Viral Pneumonia in Mice
Source: Can J Infect Dis Med Microbiol. 2024 Aug 23;2024:2017992. doi: 10.1155/2024/2017992 (PMC11364478; doi:10.1155/2024/2017992)
Supplement: Supplementary Materials — Characterization of compounds identified from MXSGD, SJZD, and MXSGD + SJZD by UPLC-Q-Orbitrap-MS, shown in Tables S1, S2, and S3. [file 2017992.f1.docx]

**Supplement information**

**Synergistic therapeutic effects and immunoregulatory mechanism of Maxing Shigan decoction combined Sijunzi decoction on** **viral pneumonia in mice**

Huimin Huang^a, #^, Huanhua Yang ^a, #^, Zurong Zhang ^a^, Yunlong Song ^a^, Li Li ^a^, Ke Li^b^, Junjie Zhang^b^, Xiaoyu Qi^b^ and Ying Wu^a*^

^a^ Liuzhou Key Laboratory of Infection Disease and Immunology, Guangxi Key Laboratory of Clinical Disease Biotechnology Research, Research Center of Medical Sciences, Liuzhou People's Hospital affiliated to Guangxi Medical University, Liuzhou 545006, Guangxi, China.

^b^ School of Life Sciences, Beijing University of Chinese Medicine, Beijing 102488, China.

^#^ Huimin Huang and Huanhua Yang contributed equally to this study.

* Correspondence author: Ying Wu (Email: aqiwuying@163.com)

Liuzhou Key Laboratory of Infection Disease and Immunology, Guangxi Key Laboratory of Clinical Disease Biotechnology Research, Research Center of Medical Sciences, Liuzhou People's Hospital affiliated to Guangxi Medical University, Liuzhou 545006, Guangxi, China

Telephone: +86-0772-2663136

ORCID-ID 0000-0002-7070-2644

Table S1 Characterization of compounds identified from MXSGD by UPLC-Q-Orbitrap-MS

| No. | t_R_  (min) | Molecular  formula | Selected ion | Theo.mass | Measured mass | Diff  (ppm) | Fragmentations (m/z) | Identified Compounds |
| --- | --- | --- | --- | --- | --- | --- | --- | --- |
| 1 | 1.44 | C_5_H_10_NO_2_ | [M+H]^+^ | 116.07061 | 116.0704 | -2.06 | 58.0655, 70.0651 | Proline |
| 2 | 3.38 | C_7_H_5_O_5_ | [M-H]^-^ | 169.01315 | 169.0143 | 7.06 | 125.0244, 101.0244 | Gallic acid |
| 3 | 5.90 | C_9_H_14_NO | [M+H]^+^ | 152.10699 | 152.1067 | -2.08 | 134.0598, 117.0696 | Phenylpropanolamine |
| 4 | 6.5 | C_9_H_14_NO | [M+H]^+^ | 152.10699 | 152.1067 | -1.78 | 134.0961, 117.0696 | Cathine |
| 5 | 7.50 | C_10_H_16_NO | [M+H]^+^ | 166.12264 | 166.1223 | -1.96 | 148.1117, 133.0883, 117.0696 | Ephedrine |
| 6 | 7.78 | C_16_H_17_O_9_ | [M-H]^-^ | 353.08671 | 353.0881 | 4.04 | 179.0562, 191.0199, 145.0507, 129.0194, 101.0243 | Neochlorogenic acid |
| 7 | 7.86 | C_10_H_16_NO | [M+H]^+^ | 166.12264 | 166.1223 | -2.06 | 148.1119  133.0882, 117.0695 | Pseudoephedrine |
| 8 | 9.65 | C_16_H_17_O_9_ | [M-H]^-^ | 355.08671 | 353.0880 | 3.61 | 191.0562 | Chlorogenic acid |
| 9 | 10.05 | C_20_H_31_N_2_O_11_ | [M+NH_4_]^+^ | 457.19224 | 475.1913 | -2.07 | 325.1120, 296.1121 | Amygdalin |
| 10 | 11.59 | C_15_H_13_O_5_ | [M^+^H]^+^ | 273.07575 | 273.0753 | -1.70 | 255.0646, 153.0180, 119.0489 | Naringenin |
| 11 | 13.42 | C_15_H_13_O_4_ | [M+H]^+^ | 257.08084 | 257.0801 | -2.95 | 137.0230 | Isoliquiritigenin |
| 11 | 13.42 | C_15_H_11_O_4_ | [M-H]^-^ | 255.06519 | 255.0663 | 4.37 | 135.0089 | Isoliquiritigenin |
| 12 | 15.19 | C_16_H_13_O_6_ | [M+H]^+^ | 301.07066 | 301.0701 | -1.98 | 285.0749, 257.0801 | Isokaempferide |
| 13 | 17.79 | C_16_H_11_O_6_ | [M-H]^-^ | 299.05501 | 299.0561 | 3.60 | 256.0697, 180.0667 | Diosmetin |
| 14 | 18.02 | C_16_H_11_O_4_ | [M-H]^-^ | 267.06519 | 267.0663 | 4.23 | 223.0459,  195.0509,  167.0349 | Formononetin |
| 15 | 20.13 | C_15_H_9_O_5_ | [M-H]^-^ | 269.04445 | 269.0455 | 4.02 | 225.0616, 181.0718, 151.0400 | Genistein |
| 16 | 27.44 | C_20_H_17_O_6_ | [M-H]^-^ | 353.10196 | 353.1031 | 3.18 | 338.0880, 175.0622 | Luteone |

Table S2 Characterization of compounds identified from SJZD by UPLC-Q-Orbitrap-MS

| No. | t_R_  (min) | Molecular  formula | Selected ion | Theo.mass | Measured mass | Diff  (ppm) | Fragmentations (m/z) | Identified Compounds |
| --- | --- | --- | --- | --- | --- | --- | --- | --- |
| 1 | 1.44 | C_5_H_10_NO_2_ | [M+H]^+^ | 116.07061 | 116.0706 | -0.22 | 70.0652, 58.0733 | Proline |
| 2 | 7.78 | C_16_H_17_O_9_ | [M-H]^-^ | 353.08671 | 353.0882 | 4.13 | 179.0563, 191.0200, 145.0507, 129.0194, 101.0244 | Neochlorogenic acid |
| 3 | 9.65 | C_16_H_17_O_9_ | [M-H]^-^ | 353.08671 | 353.0882 | 4.30 | 191.0563 | Chlorogenic acid |
| 4 | 11.57 | C_15_H_13_O_5_ | [M+H]^+^ | 273.07575 | 273.0755 | -1.03 | 153.0181, 119.0491, 107.0490 | Naringenin |
| 5 | 13.35 | C_15_H_13_O_4_ | [M+H]^+^ | 257.08084 | 257.0806 | -1.05 | 137.0231 | Isoliquiritigenin |
| 5 | 13.35 | C_15_H_11_O_4_ | [M-H]^-^ | 255.06519 | 255.0661 | 3.41 | 135.0086 | Isoliquiritigenin |
| 6 | 15.59 | C_20_H_27_O_8_ | [M-H]^-^ | 395.17004 | 395.1717 | 4.17 | 305.1245,  215.1078,  185.0972,  159.1026 | Lobetyolin |
| 7 | 17.68 | C_15_H_9_O_6_ | [M-H]^-^ | 285.03936 | 285.0406 | 4.29 | 257.0721,  151.0401 | Luteolin |
| 8 | 18.00 | C_16_H_11_O_4_ | [M-H]^-^ | 267.06519 | 267.0665 | 4.91 | 251.0411, 223.0461, 208.0545, 185.0661, 167.0352 | Formononetin |
| 9 | 20.10 | C_15_H_9_O_5_ | [M-H]^-^ | 269.04445 | 269.0457 | 4.70 | 225.0617, 181.0720, 133.0143 | Genistein |
| 10 | 23.47 | C_30_H_45_O_4_ | [M-H]^-^ | 469.33124 | 469.3323 | 2.21 | 355.2491 | 18-β-glycyrrhetinic acid |
| 11 | 23.77 | C_30_H_47_O_4_ | [M+H]^+^ | 471.34689 | 471.3463 | -1.28 | 453.3361, 435.3249 | 16α-hydroxydehydrotrametenolic acid |
| 12 | 26.25 | C_15_H_21_O_3_ | [M+H]^+^ | 249.14852 | 249.1483 | -0.89 | 232.1411, 189.0909 | Atractylenolide III |
| 13 | 26.25 | C_15_H_19_O_2_ | [M+H]^+^ | 231.13796 | 231.1378 | -0.73 | 203.1429, 185.1324 | Atractylenolide I |
| 14 | 27.40 | C_20_H_17_O_6_ | [M-H]^-^ | 353.10196 | 353.1034 | 4.05 | 338.1479, 175.0612 | Luteone |
| 15 | 27.58 | C_21_H_21_O_4_ | [M-H]^-^ | 337.14344 | 337.1449 | 4.43 | 243.0626 | Licochalcone A |
| 16 | 28.23 | C_21_H_17_O_6_ | [M-H]^-^ | 365.10196 | 365.1033 | 3.75 | 307.0231, 295.0673, 251.0410, 207.0512 | Glycyrol |

Table S3 Characterization of compounds identified from MXSGD+SJZD by UPLC-Q-Orbitrap-MS

| No. | t_R_  (min) | Molecular  formula | Selected ion | Theo.mass | Measured mass | Diff  (ppm) | Fragmentations (m/z) | Identified Compounds |
| --- | --- | --- | --- | --- | --- | --- | --- | --- |
| 1 | 1.44 | C_5_H_10_NO_2_ | [M+H]^+^ | 116.07061 | 116.0702 | -3.11 | 58.0653, 70.0650 | Proline |
| 2 | 5.88 | C_9_H_14_NO | [M+H]^+^ | 152.10699 | 152.1065 | -2.98 | 134.0960, 117.0695 | Phenylpropanolamine |
| 3 | 6.48 | C_9_H_14_NO | [M+H]^+^ | 152.10699 | 152.1067 | -2.58 | 134.0960, 117.0695 | Cathine |
| 4 | 7.57 | C_10_H_16_NO | [M+H]^+^ | 166.12264 | 166.1221 | -3.34 | 148.1116, 133.0882, 117.0695 | Ephedrine |
| 5 | 7.81 | C_16_H_17_O_9_ | [M-H]^-^ | 353.08671 | 353.0880 | 3.69 | 179.0562, 191.0199, 145.0506, 135.0299, 129.0194, 101.0244 | Neochlorogenic acid |
| 6 | 7.86 | C_10_H_16_NO | [M+H]^+^ | 166.12264 | 166.1221 | -2.97 | 133.0885, 117.0571 | Pseudoephedrine |
| 7 | 9.07 | C_9_H_7_O_2_ | [M-H]^-^ | 147.04406 | 147.0452 | 2.67 | 102.9487, 90.9334 | Trans-cinnamic acid |
| 8 | 9.66 | C_16_H_17_O_9_ | [M-H]^-^ | 353.08671 | 353.0880 | -2.5 | 191.0561 | Chlorogenic acid |
| 9 | 10.12 | C_20_ H_31_N_2_O_11_ | [M+NH_4_]^+^ | 475.1924 | 475.1909 | -2.77 | 325.1118, 296.1118 | Amygdalin |
| 10 | 11.57 | C_15_H_11_O_5_ | [M-H]- | 271.06010 | 271.0614 | 4.69 | 151.0035, 119.0501, 107.0139 | Naringenin |
| 11 | 13.41 | C_15_H_13_O_4_ | [M+H]^+^ | 257.08084 | 257.0797 | -4.37 | 137.0227 | Isoliquiritigenin |
| 11 | 13.41 | C_15_H_11_O_4_ | [M-H]^-^ | 255.06519 | 255.0660 | 3.05 | 135.0802 | Isoliquiritigenin |
| 12 | 15.20 | C_16_H_13_O_6_ | [M+H]^+^ | 301.07066 | 301.0698 | -2.99 | 285.0748, 257.0800 | Isokaempferide |
| 13 | 15.61 | C_20_H_27_O_8_ | [M-H]^-^ | 395.17004 | 395.1713 | 3.09 | 305.1241,  215.1077,  185.0971,  159.0814 | Lobetyolin |
| 14 | 17.72 | C_15_H_9_O_6_ | [M-H]^-^ | 285.03936 | 285.0403 | 3.44 | 257.0719,  151.0399, | Luteolin |
| 15 | 17.80 | C_16_H_11_O_6_ | [M-H]^-^ | 299.05501 | 299.0561 | 3.50 | 284.0324, 256.0696, 180.0666 | Diosmetin |
| 16 | 18.02 | C_16_H_11_O_4_ | [M-H]^-^ | 267.06519 | 267.0662 | 3.77 | 251.0409,  223.0459,  208.0544,  195.0510,  167.0350 | Formononetin |
| 17 | 20.14 | C_15_H_9_O_5_ | [M-H]^-^ | 269.04445 | 269.0455 | 3.91 | 225.0615, 181.0717, 151.0399 | Genistein |
| 18 | 23.47 | C_30_H_45_O_4_ | [M-H]^-^ | 469.33124 | 469.3313 | 0.13 | 425.3421 | 18-β-glycyrrhetinic acid |
| 19 | 23.88 | C_30_H_47_O_4_ | [M+H]^+^ | 471.34689 | 471.3455 | -2.90 | 453.3342, 435.3251, | 16α-hydroxydehydrotrametenolic acid |
| 20 | 24.26 | C_30_H_47_O_3_ | [M+H]^+^ | 455.35197 | 455.3507 | -2.71 | 437.3403, 313.2365, 295.2260, | Dehydrotrametenolic acid |
| 21 | 26.38 | C_15_H_21_O_3_ | [M+H]^+^ | 249.14852 | 249.1476 | -3.52 | 232.1372, 189.0903 | Atractylenolide III |
| 21 | 26.38 | C_15_H_19_O_2_ | [M+H]^+^ | 231.13796 | 231.1372 | -3.37 | 203.1423, 185.1320, 161.0592, 149.0229 | Atractylenolide I |
| 22 | 27.44 | C_20_H_17_O_6_ | [M-H]^-^ | 353.10196 | 353.1031 | 3.10 | 338.1468, 175.0246 | Luteone |
| 23 | 27.67 | C_21_H_21_O_4_ | [M-H]^-^ | 337.14344 | 337.1447 | 3.61 | 281.0878, 243.0619 | Licochalcone A |
| 24 | 28.27 | C_21_H_17_O_6_ | [M-H]^-^ | 365.10196 | 365.1029 | 2.58 | 307.0668, 295.0675, 251.0408, 207.0509 | Glycyrol |
